# Maternal High‐Fat Diet, During Preimplantation or Gestation/Lactation, Alters Mouse Offspring Metabolic, Molecular and Locomotor Phenotypes

**DOI:** 10.1155/jnme/5286440

**Published:** 2026-09-30

**Authors:** Irene Peral-Sanchez, Eda Sezer, Diego A. Ojeda, Philip C. Calder, Patrick C. McHugh, Rachel Morris, Jeanette L. Norman, Jon Ward, Tom P. Fleming, Judith J. Eckert, Neil R. Smyth, Sandrine Willaime-Morawek

**Affiliations:** ^1^ Faculty of Medicine Southampton General Hospital University of Southampton Southampton SO16 6YD UK southampton.ac.uk; ^2^ Medical Sciences Faculty of Medicine University of Bakırçay İzmir Türkiye; ^3^ Department of Ophthalmology Bascom Palmer Eye Institute University of Miami Miller School of Medicine Miami Florida USA miami.edu; ^4^ Centre for Biomarker Research Department of Pharmacy School of Applied Sciences University of Huddersfield Huddersfield HD1 3DH UK hud.ac.uk; ^5^ School of Biological Sciences University of Southampton Southampton General Hospital Southampton SO16 6YD UK southampton.ac.uk

**Keywords:** DOHaD, maternal diet, nutrition

## Abstract

Maternal nutrition during offspring development influences the offspring physiology and risk of long‐term chronic disease. Here, we investigate the effects of maternal high‐fat diet during distinct developmental windows (preimplantation alone, EmbHFD; or throughout gestation and lactation, HFD) on adult offspring metabolic, molecular and motor behavioural outcome. Our mouse model, utilising healthy nonobese MF1 mothers, induced distinct offspring outcomes dependent upon dietary duration with some sex‐specific effects. EmbHFD offspring had high serum leptin (females), elevated systolic blood pressure (SBP), low locomotory activity and diminished motor coordination. Similarly, HFD offspring not only had raised serum leptin, high SBP and poor motor coordination but also experienced excess weight gain, deficits in energy metabolism, reduced relative brown adipose tissue (BAT) mass (females) and increased muscle lipid accumulation. Transcriptomic profiling of HFD female offspring revealed upregulation of thermogenic and lipid‐handling genes (e.g., *Ucp1* and *ApoE*) in BAT alongside downregulation of mitotic regulators, suggesting impaired regenerative capacity. Collectively, these findings demonstrate, first, the risk to offspring of even transient maternal fatty diet without maternal obesity. Second, they highlight the sensitivity of periconceptional nutrition on the emergence of offspring multisystem metabolism and physiology, supporting the Developmental Origins of Health and Disease (DOHaD) concept.

**Key Points**
• Maternal high‐fat diet during early development, even if transient and not associated with obesity, can cause long‐term physiological changes in offspring.
• Offspring exposed to a high‐fat diet during preimplantation (EmbHFD) showed elevated blood pressure, reduced motor coordination and sex‐specific increases in serum leptin.
• Offspring exposed to a high‐fat diet throughout gestation and lactation (HFD) exhibited additional metabolic impairments, including excess weight gain, reduced brown adipose tissue mass (in females) and increased muscle lipid accumulation.
• Transcriptomic analysis of brown adipose tissue in HFD female offspring revealed altered expression of genes involved in thermogenesis and cell division, suggesting compromised tissue regeneration and metabolic regulation.

## 1. Introduction

The worldwide increase in non‐communicable diseases (NCDs), including obesity, cardiovascular disease and Type 2 diabetes, is closely associated with the rising prevalence of high‐fat diets (HFDs), especially among women of reproductive age [1–3]. Maternal obesity significantly contributes to metabolic dysfunction in children, increasing their risk of obesity, insulin resistance and neurodevelopmental disorders in adulthood [4–7]. Moreover, across many animal models, maternal obesity has been shown to induce cardiometabolic and neurodevelopmental diseases in offspring [8–11]. In a wider context, the implications of maternal overnutrition and obesity on offspring health fit within the Developmental Origins of Health and Disease (DOHaD) hypothesis where prenatal exposure to diverse environmental conditions can impact next‐generation health outcomes [12, 13]. Moreover, within DOHaD, the periconceptional period of development prior to embryo implantation has been recognised as a vulnerable period when adverse programming of offspring health can take place [14, 15] associated with epigenetic and signalling pathways [16–18].

Maternal obesity is a multifactorial condition whereby effects on the developing offspring might be transmitted through diverse pathways mediated by endocrine, metabolic, dietary, inflammatory or epigenetic status. To pinpoint mechanistic processes, new models are required to discriminate between these factors. For example, within a healthy, normal‐weight female, does transient consumption of a HFD affect her developing offspring? Emerging evidence suggests that poor diet alone, in the absence of maternal obesity, uniquely shapes developmental programming, affecting energy metabolism and neurogenesis [14, 19, 20].

In the current study, we extend our model of maternal HFD consumption independent of obesity, comparing effects of exposure during only the preimplantation period or throughout gestation and lactation, on offspring phenotype. This complements our earlier study focussing on neurodevelopmental outcomes [19]. Here, we hypothesise that in non‐obese dams, a transient maternal over‐nutrient stress during preimplantation alters developmental programming in a sex‐specific way through differing metabolic and physiological phenotypes and related behaviours, when compared to prolonged maternal over‐nutrient stress, spanning gestation and lactation. This study suggests that changes in the BAT transcript expression are the mechanistic link.

## 2. Methods

### 2.1. Ethical Approval

Experimental procedures were conducted using protocols approved by, and in accordance with, the UK Home Office Animal (Scientific Procedures) Act 1986 and local ethics committee at the University of Southampton under the Licence PPL PAFA91AD and approval ERGOII 53180. The investigators understand the ethical principles under which the journal operates and their work complies with its animal ethics checklist and the ARRIVE guidelines.

### 2.2. Animals and Diet Groups

The normal fat diet (NFD) was RM1 (rat and mouse no. 1 maintenance, 801,151) obtained from SDS (Special Diet Services, Essex, UK) and containing 7.5% kcal fat, 17.5% kcal protein, and 75% kcal carbohydrate. The HFD was also obtained from SDS (824,053) and contained 45% kcal fat, 20% kcal protein, and 35% kcal carbohydrate. The caloric composition of the two diets was 3.53 kcal/g (NFD) and 4.54 kcal/g (HFD).

7–10‐week‐old outbred female MF1 mice were bred in‐house (Biomedical Research Facility, University of Southampton) and housed in a 0700–1900 light/dark cycle at constant temperature (22 ± 2°C) with *ad libitum* access to food (NFD) and water. Fifteen 7–14‐week‐old MF1 males fed NFD were used for natural mating. The morning of plug confirmation (G0.5), each dam was randomly assigned to a diet group, ensuring equal group sizes, and individually housed: CFD (NFD during the whole gestation and lactation period), EmbHFD (HFD until G3.5, NFD thereafter), and HFD (HFD throughout gestation and lactation) totalling 26 litters (Figure 1A). The litter was normalised to three males and three females per mother at P3 (3 days after birth). After weaning at P21 (21 days after birth), male and female siblings were housed together in each litter and fed NFD until they were 15 weeks of age. A smaller cohort was also generated, with mothers culled at G3.5 for serum collection comprising CFD 8 females and EmbHFD 8 females. Researchers were blind to the groups for the offspring animals but not the mothers (diet looked different in the cages).

**FIGURE 1:**
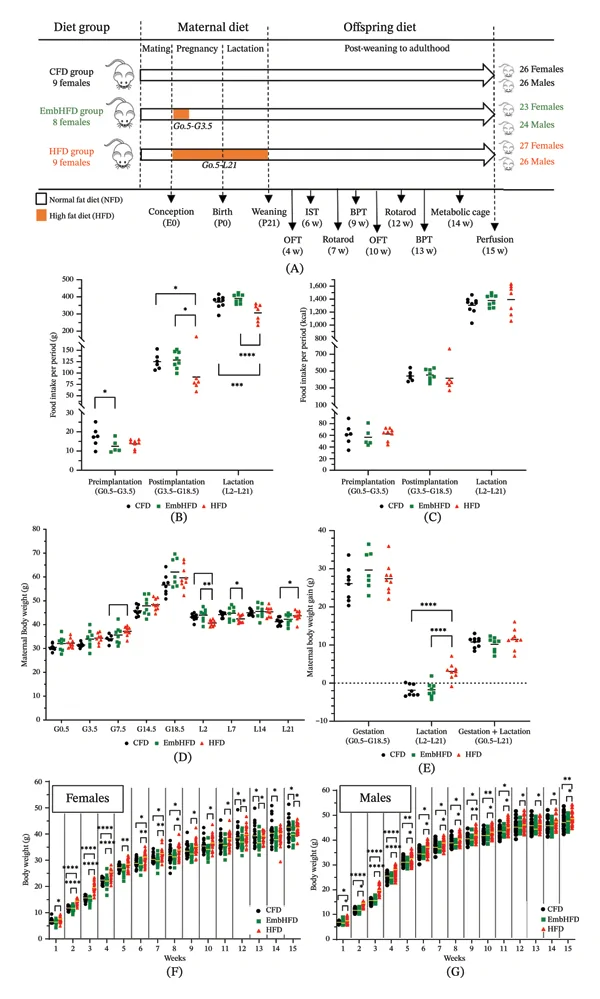
High‐fat diet during gestation and lactation does not induce higher calorie intake nor obesity, validating the maternal high‐fat diet model in the absence of obesity. (A). Experimental design: MF1 mice were divided into three diet groups, CFD, EmbHFD, and HFD and were exposed to NFD and/or HFD during their gestation and lactation. After weaning (L21/P21), the offspring physiological tests were performed as follows: open field test (OFT, 4 and 10 weeks), inverted screen test (IST, 6 weeks), rotarod (7 and 12 weeks), blood pressure test (BPT, 9 and 13 weeks) and metabolic cage (14 weeks). Finally, offspring were perfused at 15 weeks, and tissues were collected. A smaller and shorter cohort was also generated, with mothers culled at G3.5 for serum collection; it had only two groups, CFD 8 females and EmbHFD 8 females. (B, C) Food consumption during preimplantation (G0.5–3.5), postimplantation (G3.5–G18.5) and lactation (L2–L21), represented as mass (B, g) or energy (C, kcal) consumed during each period. Number of mothers: 6–9 CFD, 5–8 EmbHFD and 6–8 HFD. (D) Mothers’ body weight during gestation (G0.5, G3.5, G7.5, G14.5, G18.5) and lactation (L2, L7, L14, L21). Number of mothers: 6–9 CFD, 7‐8 EmbHFD and 8‐9 HFD. (E) Mothers’ body weight gain during gestation (G0.5–G18.5), lactation (L2–L21) or both (G0.5–L21). The body weight gain was assessed by calculating the difference between the end and start dates as specified. Number of mothers: 8‐9 CFD, 7‐8 EmbHFD and 9 HFD. (F–G) Female (F) and male (G) offspring body weight over the 15 weeks of life. E = embryonic; P = postnatal, related to the offspring; G = gestation, L = lactation, related to the mother. CFD data (black circles), EmbHFD data (green squares) and HFD data (red triangles) were analysed by multilevel random effects regression and shown as dot plots with mean (black in A–E or yellow in F–G horizontal lines) ^∗^ *p* < 0.05, ^∗∗^ *p* < 0.01, ^∗∗∗^ *p* < 0.001 and ^∗∗∗∗^ *p* < 0.0001 between diet groups.

### 2.3. Food Intake and Body Weight Measurement

Maternal food intake was determined during gestation and lactation every 2 or 3 days. Food consumption during preimplantation (G0.5–3.5), postimplantation (G3.5–G18.5) and lactation (L2–L21) was measured and analysed as mass (g) or energy (kcal) consumed during each period.

Maternal body weight was measured on plug day (G0.5), as well as on G3.5, G7.5. G14.5 and G18.5 during gestation and during lactation on L2, L7, L14 and L21.

### 2.4. Behaviour Tests

Inverted screen test (IST): IST was performed by offspring at age 6 weeks [21, 22]. Animals were acclimatised to the test room for 30 min to minimise stress. The screen was a 43‐cm^2^ wire mesh with 12‐mm^2^ squares and a 1‐mm wire, surrounded by a 4‐cm wood border preventing access to the other side of screen. The mouse was placed in the middle of the screen, the screen was inverted 60 cm above a protective bedding and time was recorded until the mouse fell (up to 15 min; 900 s). The test was performed three times with a 30‐min rest between each trial.

Rotarod test: Offspring mice were tested at age 7 and 12 weeks on the Ugo Basile 7650 accelerating mouse Rotarod (Ugo Basile®, Italy) [23, 24]. Animals were acclimatised to the test room for 30 min to minimise stress. Prior to the test day, animals performed 2 days of training, learning to walk on the rod rotating at 4 rpm for up to 120 s, three times, with a 10‐min rest between trials. On the test day, animals were acclimatised to the test room for 30 min to minimise stress. Once the mice were on the rod, it accelerated from 4 to 40 rpm for a maximum of 300 s and the time until the mouse fell was recorded. The test was completed three times with 10‐min rest intervals in between.

### 2.5. Open Field Test (OFT)

Offspring were tested in an open field box (28.5 cm × 28.5 cm × 28.5 cm), for 10 min at age 4 and 10 weeks, after a 30‐min room habituation. Animal tracking was performed using the ENV250 Test Software (MED Associates, Inc. Vermont) and sessions were video‐recorded. Between each test, the open field was cleaned with 70% ethanol and air‐dried. The animal was placed in the middle of the platform. The total distance covered, average velocity, vertical counts and times, jump counts, time spent in the central zone and frequency of entering the central zone were signs of exploratory activity. Conversely, the resting time and time spent in the outer zone were signs of non‐explorative behaviour and higher anxiety, respectively. 10‐min testing sessions were analysed as two periods of 5 min.

### 2.6. Systolic Blood Pressure (SBP) Measurement

SBP was measured in offspring at age 9 and 13 weeks by a tail‐cuff plethysmography using a non‐invasive blood pressure monitor (NIBP‐8, Columbus Instruments, Columbus, OH, USA). SBP was measured in a preheated room at 25°C–28°C to encourage vasodilatation. Mice were habituated to the room for 30 min and to the tail‐cuff apparatus for 5 min before testing. Mice were restrained in a ventilated acrylic tube while their tails were threaded through the cuff. Five SBP recordings with clear waveforms were taken per mouse, and the mean of the three middle readings was recorded. If this was not achieved after 20 min, the mouse was released and allowed to recover before proceeding again. Heart rate was monitored as an indicator of stress, and readings were not taken if the heart rate was over 600 beats/min.

### 2.7. Metabolic Indirect Calorimetry

The CaloBox (PhenoSys GmbH, Germany) was used to perform indirect calorimetry in offspring mice at age 14 weeks. Analysis conditions (temperature, pressure, and gas mixture) were stabilised prior to placing the mouse in the metabolic cage. Animals were placed in the metabolic cage at 2–3 pm to acclimatise overnight. Measurements began at 7 am for 24 h and were taken every 20 s; measurements comprised energy expenditure (EE, cal/min), CO_2_ production (mL/min), O_2_ used (mL/min) and respiratory exchange ratio (RER, volume of CO_2_ produced/O_2_ consumed). Readings were recorded by the software (PhenoSys GmbH, Germany), and the means for 2 h and 12 h intervals were calculated for each mouse.

### 2.8. Perfusion and Tissue Collection

After weighing, animals were anaesthetised (sodium pentobarbitone, Iso‐Vet®, UK), via intraperitoneal injection. A saline solution (0.9 g NaCl/100 mL) was perfused with maintained pressure until the liver was clear. The animals were dissected, and serum and tissues (muscle, spleen, ovary, uterus, testis, kidneys, liver, fat pads, brain and pancreas) were collected for both molecular and histological analyses. Liver and BAT were weighed prior to preservation.

### 2.9. Serum Fatty Acid Composition

Serum from pregnant mice at G3.5, from dams after weaning and from offspring at age 15 weeks were analysed as previously described [25]. Total lipids were first extracted into chloroform:methanol (2:1 vol/vol) and then separated into triacylglycerols (TAGs), cholesteryl esters (CEs), phosphatidylcholines (PCs), and non‐esterified fatty acids (NEFAs) by solid phase extraction. Fatty acid methyl esters of each lipid fraction were produced by heating in sulphuric acid containing 2% methanol. Fatty acid methyl esters were separated by gas chromatography (Hewlett Packard 6890; Agilent, UK) using the conditions described elsewhere (Fisk et al., 2014) [25]. Data were analysed using HPChemStation software (Agilent, UK); an internal standard was added to each sample at the start of the processing, and so each fatty acid is expressed as μg/mL serum.

### 2.10. Serum Leptin Concentration

Serum from pregnant mice at G3.5, from dams after weaning and offspring at age 15 weeks were analysed using the Enzo Life Sciences Leptin ELISA kit (ENZO Life, ADI‐900‐019A, Germany) following the manufacturer’s protocol. Serum samples were diluted to the previously tested optimal concentration (1:25) and run in duplicate. Readings were taken at 450 nm using an Infinite® 200 pro plate reader (TECAN, Switzerland).

### 2.11. Muscle Histology

Gastrocnemius muscles were collected from offspring used in the Calobox testing where possible. Right legs were slow‐frozen in isopentane and OCT following a published protocol [26] and stored at −80°C. Transverse sections at the mid‐belly of the muscle were cryostat (Leica Biosystems, Germany)‐sectioned at 10 μm at −24°C. Quality and orientation of muscle sections were assessed by Mayers haematoxylin (Sigma‐Aldrich, USA) staining for 1 min and observed. Sections were placed on Superfrost Plus slides (Thermo Fisher Scientific), 8 per slide, 14 slides per muscle and stored at −20°C. Slides were brought to room temperature for 5 min, washed in water and then 70% ethanol, and incubated in Sudan Black B solution (Merck, 1,999,664, Germany) for 90 s, rinsed in 70% ethanol and running water; nuclei were counterstained with nuclear fast red (Sigma‐Aldrich, 6409‐77‐4, UK) for 30 s and samples were washed in water. Slides were mounted in Kaiser’s Glycerine jelly (Merck Millipore, 10,924, Germany) with a coverslip (Thermo Fisher Scientific), stored at 4°C and analysed within 2 weeks. Slides were examined on a DM 5000B microscope (Leica, Germany) with camera (Leica, DFC 7000t camera, Germany) using LAS‐X software (Leica UK Ltd, Milton Keynes). Fiji Image *J* was used to quantify various parameters of stained muscle. Five random pictures were taken from each muscle section, and three muscle sections were analysed per animal to determine the number of cells and proportion positively stained.

### 2.12. RNA Isolation and cDNA Synthesis

Total RNA was isolated from snap‐frozen BAT from adult offspring using RNeasy Lipid Tissue mini kit (Qiagen, UK) according to the manufacturer’s instructions. Isolated RNA was quantified using a Nanodrop ND‐1000 spectrophotometer (LabTech UK), and samples with adequate RNA concentration (A260/A280 ≥ 1.8) and purity (A230/A260 ≥ 2.0) were selected for RNA‐seq and reverse transcription.

### 2.13. RNA Sequencing

A total of 48 offspring BAT RNA samples (8 samples per diet group and sex) were analysed that had an RNA integrity number (RIN) > 8. RNA‐seq was performed using the Illumina® platform, and the raw data were set up using R. Data with adjusted *p* value above 0.05 and with log2fold values between −1 and 1 were excluded to avoid false discoveries. Once filtered, data were split into expression upregulated (log2fold positive) or downregulated (log2fold negative) relative to CFD, and each group was analysed in GeneOntology (GO) and verification String (string‐db.org) and Kyoto Encyclopaedia of Genes and Genomes (KEGG) [27].

### 2.14. qPCR

One microgram of RNA from each sample was reverse‐transcribed using Precision Reverse Transcription Premix (PrimerDesing Ltd, UK) following the manufacturer’s protocol. All qPCR reactions used a CFX96 Real‐Time System/C1000 Thermal Cycler (BioRad, UK), analysed with CFX Manager® software version 3.1 (Bio‐Rad, UK). All reactions were run in duplicate, using low‐profile, non‐skirted, clear 96‐well plates (E1403‐0200, Starlab, UK), and each reaction had a no‐template and non‐reverse transcript controls. The reactions were performed using 10 μL of SYBR‐green 2 x PrecisionPlus® MasterMix (Primerdesign UK), 2 μL of forward and reverse primers (1.0 μM), 2 μL of nuclease‐free water and 4 μL of cDNA sample. Lyophilised primers (Merk, Sigma‐Aldrich, UK) were reconstituted to 100‐μM stock solutions and 10‐μM working solutions in nuclease‐free water. The amplification programme included enzyme activation at 95°C for 5 min, denaturation at 95°C for 15 s, annealing at 60°C for 60 s, for 40 cycles and a final extension step at 72°C for 10 min. Melting curves were generated for each sample to verify amplification specificity by fluorescence detection between 60°C and 90°C at 0.1°C steps.

A set of 9‐candidate reference genes were run on a separate plate (*n* = 7/sex and diet group). Reference gene stability was determined using Biogazelle qbase + 3.3 (Biogazelle, Belgium), a relative quantification software. The stability of the different candidate reference genes was based on algorithm geNorm *M* and *V* values. For BAT qPCR, the optimal normalisation factor was calculated as the geometric mean of reference targets Gapdh, Pgk1, Ppib and Sdha. Data analysis was performed using the comparative CT method (2^−ΔΔCT) to evaluate relative gene expression following statistical analysis [28] using ΔCT = average gene *C* (*t*) − average HKs *C* (*t*), ΔΔCT = average Δ*C* (*t*) − average Δ*C* (*t*) of control group. To assess primer efficiency, a standard curve for each gene was generated. Efficiency of the primer between 95% and 110%, indicates that the assay is robust, and the primers are efficient [29].

### 2.15. Statistical Analysis

The sample size was determined based on prior studies using comparable models and outcome measures, which reported sufficient statistical power with similar group sizes, thereby informing our choice to ensure robust and reproducible detection of biologically relevant effects. No animal was excluded from the study.

A mixed effects model was used to statistically analyse individual mother and offspring data as used previously [19]. Data were standardised to *Z*‐scores to aid comparability across outcomes, and normality was evaluated on model residuals, calculated automatically by the syntax. Different parameters were taken into account using the mixed effects model: maternal diet group as fixed effect, alongside litter size, sex and body weight from individual animals. Litter effects were controlled either by using only one male and one female per litter for experimental analysis or, if more than one male or female were used, by incorporating into the multilevel random effects regression model. This takes into account the hierarchical nature of the data sets with between‐mother and within‐mother variation and different parameters measured from individual animals. Thus, differences identified between treatment groups are independent of maternal origin of litter, litter size and body weight. Similarly, data from mothers were analysed using the mixed effects model with diet group and body weight as parameters.

The trapezoidal rule was used to determine the area under the curve (AUC) for each animal, removing the baseline, using GraphPad Prism Version 9.4.1; the analysis of the AUC values was then carried out on the mixed effects model.

SPSS (version 27.0) was used for statistical analysis of data, except for fatty acid content where GraphPad Prism version 9.4.1 was used and normality was assessed through a Shapiro–Wilk test. Each fatty acid of each phase at G3.5 was analysed separately with a *t*‐test. One‐way ANOVA was used at L21 and two‐way ANOVA for offspring.

The data that support the findings of this study are openly available in the Pure University of Southampton at https://pure.soton.ac.uk/, reference numbers https://doi.org/10.5258/SOTON/D2904 and https://doi.org/10.5258/SOTON/D3117.

## 3. Results

### 3.1. HFD and EmbHFD Treatments Do Not Cause Obesity in Mothers or Offspring

Maternal food consumption was measured during gestation and lactation. Pregnant dams ate less HFD (in mass) during preimplantation (G0.5–G3.5), postimplantation (G3.5–G18.5) and lactation (L2–L21) affecting both EmbHFD and HFD groups (Figure 1B). However, since the diets were not isocaloric, energy ingested was not different between treatments in each period (Figure 1C). Maternal weight was also similar between groups through to the end of lactation, although HFD dams were heavier than CFD at G7.5 and L21 and lighter than CFD at L2 and EmbHFD at L2 and L7 (Figure 1D). Dams were assessed for index of obesity [30–32]; at no time points did EmbHFD or HFD dams reach 20% IOD, confirming that none were classified as obese [19, 33, 34]. Maternal weight gain was also not different between groups during gestation although HFD dams had greater weight gain than other groups during lactation; however, weight gain over combined gestation and lactation was not different (Figure 1E). Offspring weight over 15 weeks, in contrast, revealed males and females of mothers fed HFD to be heavier than offspring of those fed CFD in most weeks; EmbHFD offspring were lighter than HFD offspring and similar to CFD offspring most weeks (Figure 1F,G). However, according to IOD calculation, no offspring were obese [19, 33, 34]. Collectively, these data confirm that effects from our model are mediated through diet rather than maternal obesity.

### 3.2. Serum Fatty Acids Are Transiently Increased in EmbHFD Mothers While Leptin Levels Are Increased in EmbHFD and HFD Mothers and Offspring

Fatty acid composition was analysed by chromatography in maternal serum of EmbHFD and CFD groups at G3.5, upon completion of the preimplantation dietary treatment. Several fatty acids were increased in the EmbHFD group compared to CFD: 14:0, 18:0, 18:1*n* − 9, 18:3*n* − 6 and 20:1*n* − 9 in the TAG fraction (Figure 2A, file S1), 20:1*n* − 9, 20:2*n* − 6, 20:4*n* − 6, 20:5*n* − 3 and 22:5*n* − 3 in the CE fraction (Figure 2B, file S1) and 14:0, 18:3*n* − 3, 20:0, 20:1*n* − 9, 20:2*n* − 6, 20:5*n* − 3 and 24:0 in the PC fraction (Figure 2C, file S1). No differences were found in the NEFA fraction (file S1). Further analysis at the end of lactation, however, found no changes in maternal fatty acid composition in any fraction between the three dietary groups (file S1). Fatty acid composition was also analysed in 15‐week‐old offspring; the only difference was for 20:0 in the PC fraction, which was higher in EmbHFD and HFD males compared to CFD males (file S1). Thus, changes in maternal fatty acid composition occur transiently after switch to high‐fat diet (EmbHFD) but are not sustained through to the end of lactation either after continued treatment (HFD) or after return to the control diet (EmbHFD). Offspring also show minimal effects on fatty acid levels across diet groups.

**FIGURE 2:**
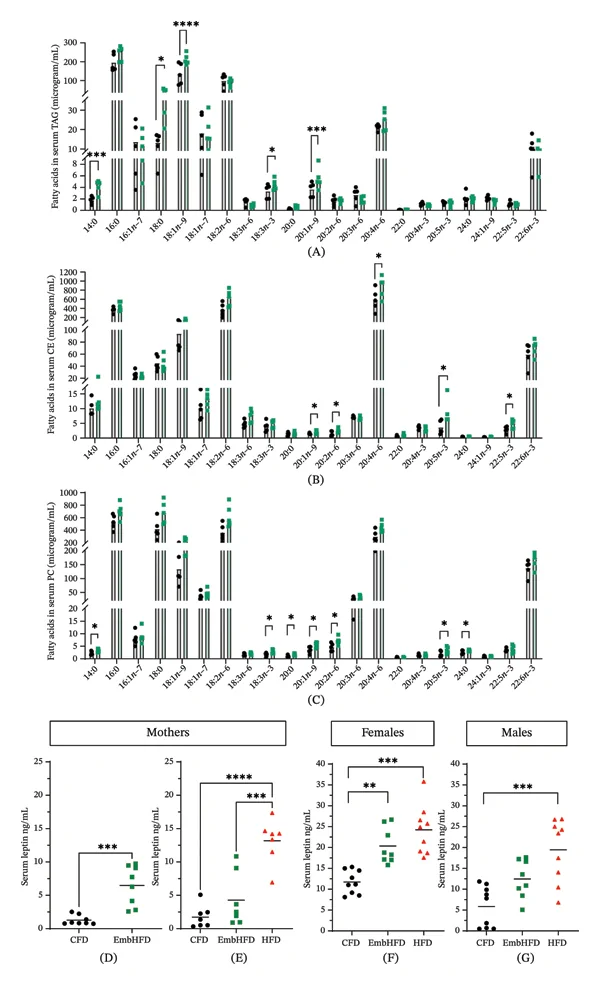
Maternal high‐fat diet increases some maternal serum fatty acid concentration and increases maternal and offspring serum leptin concentration. Fatty acid content of lipids from serum TAG (A), CE (B), and PC (C) of mothers at G3.5. Means also provided in Supporting File S1. Number of mothers: 5 CFD, 5 EmbHFD. CFD data (black circles and grey bars) and EmbHFD data (green squares and green bars) were analysed by multilevel random effects regression and shown as bar graphs with individual data. Serum leptin concentration (ng/mL) in mothers culled at G3.5 (D), mothers culled at L21 (E), and female (F) and male (G) offspring culled at 15 weeks. Number of mothers: 8 CFD, 8 EmbHFD (D, G3.5), 7 CFD, 7 EmbHFD, 7 HFD (E, L21), 9 CFD, 8 EmbHFD, 9 HFD (F, females and G, males at 15 weeks, 1 male and 1 female per mother). CFD data (black circles), EmbHFD data (green squares) and HFD data (red triangles) were analysed by multilevel random effects regression and shown as dot plots with mean. ^∗^ *p* < 0.05, ^∗∗^ *p* < 0.01, ^∗∗∗^ *p* < 0.001 and ^∗∗∗∗^ *p* < 0.0001 between diet groups.

Maternal serum leptin concentration was increased at G3.5 (EmbHFD) and at L21 (HFD) but returned to control level at L21 in EmbHFD (Figure 2D,E). Offspring, however, showed elevated serum leptin levels at age 15 weeks in EmbHFD females and HFD females and males despite themselves never consuming a HFD (Figure 2F,G).

### 3.3. EmbHFD and HFD Offspring Exhibit Raised Systolic Blood Pressure While Only HFD Changes Offspring Energy Metabolism

SBP, measured at 9 and 13 weeks, increased in EmbHFD and HFD offspring males and females relative to CFD, particularly as maturity was reached (Figure 3A–D).

**FIGURE 3:**
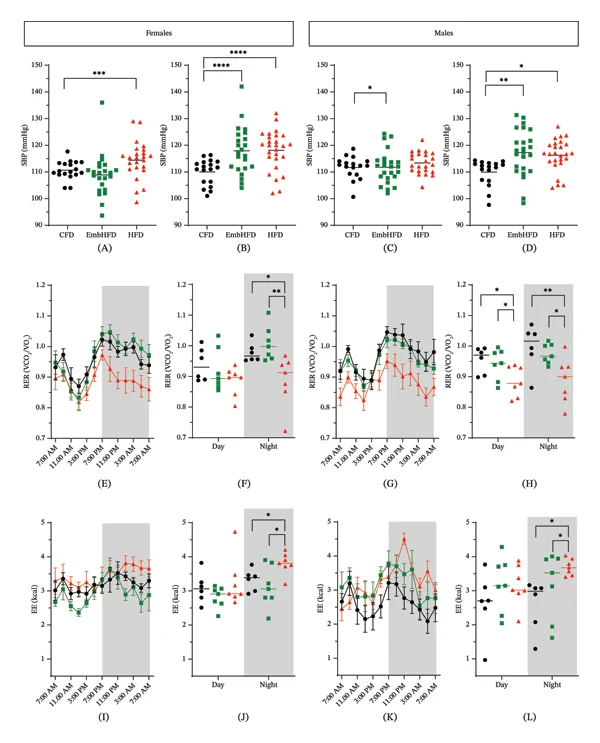
Maternal high‐fat diet increases blood pressure and energy expenditure and decreases the respiratory exchange ratio in offspring. Blood pressure in female (A, B) and male (C, D) offspring at 9 weeks (A, C) and 13 weeks (B, D). Number of female offspring: 21 CFD, 23 EmbHFD, 27 HFD, number of male offspring: 20 CFD, 24 EmbHFD, 26 HFD, number of mothers: 6 CFD, 8 EmbHFD and 9 HFD. Respiratory exchange ratio (RER) in female (E, F) and male (G, H) offspring at 14 weeks, over time (E, G) or per 12 h (F, H). Number of female offspring: 6 CFD, 7 EmbHFD, 7 HFD, number of male offspring: 6 CFD, 7 EmbHFD, 7 HFD, number of mothers: 6 CFD, 7 EmbHFD and 7 HFD. Energy expenditure (EE) in female (I, J) and male (K, L) offspring at 14 weeks, over time (I, K) or per 12 h (J, L). Number of female offspring: 6 CFD, 7 EmbHFD, 7 HFD, number of male offspring: 6 CFD, 7 EmbHFD, 7 HFD, number of mothers: 6 CFD, 7 EmbHFD and 7 HFD. CFD data (black circles), EmbHFD data (green squares) and HFD data (red triangles) were analysed by multilevel random effects regression and shown as dot plots with mean. Day as 7 AM–6.59 PM; night as 7 PM–6.59 AM (shaded area). ^∗^ *p* < 0.05, ^∗∗^ *p* < 0.01, ^∗∗∗^ *p* < 0.001 and ^∗∗∗∗^ *p* < 0.0001 between diet groups.

RER and EE were assessed at age 14 weeks in offspring using the metabolic cage. RER decreased in HFD females and males at night and in HFD males during the day, compared to CFD, while EmbHFD offspring were unaffected (Figure 3E–H). EE increased in HFD females and males at night, compared to CFD, with no changes evident in day or in EmbHFD (Figure 3I–L). Thus, the duration of maternal HFD differentially influences offspring physiological activity, with SBP being more sensitive, affecting EmbHFD.

### 3.4. HFD Female Offspring Have Reduced Relative BAT Mass While Both EmbHFD and HFD Females Exhibit More BAT Metabolic Gene Expression Changes Than Males

Relative BAT mass (BAT/body weight %), a key factor in thermogenesis and EE [35], was significantly reduced in EmbHFD but not HFD offspring females, and not affected in males, at age 15 weeks (Figure 4A,B).

**FIGURE 4:**
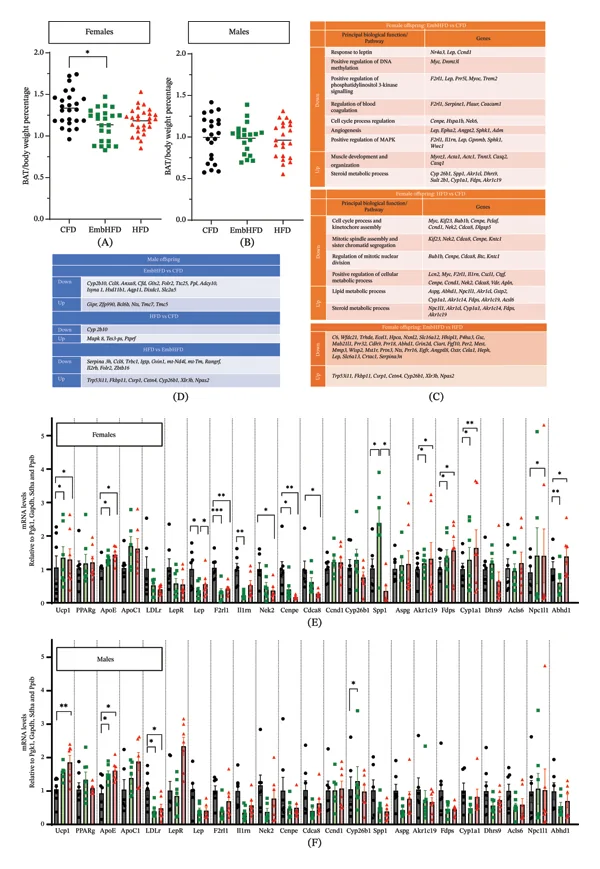
Maternal high‐fat diet decreases BAT to body weight percentage in female offspring and alters BAT gene expression. Female (A) and male (B) offspring BAT/body weight percentage at 15 weeks. CFD data (black circles), EmbHFD data (green squares) and HFD data (red triangles) were analysed by multilevel random effects regression and shown as dot plots with mean. Number of female offspring: 25 CFD, 23 EmbHFD, 27 HFD, number of male offspring: 26 CFD, 24 EmbHFD, 26 HFD, number of mothers: 9 CFD, 8 EmbHFD and 9 HFD. RNA‐seq analysis of female (C) and male (D) offspring BAT tissues at 15 weeks. Down‐ and upregulated significant genes according to our pre‐established parameters (adjusted *p* value < 0.05 and 1 ≥ log fold ≤ −1). Female offspring EmbHFD vs CFD and HFD vs CFD comparisons are selected principal biological function/pathways of interest and related genes significantly regulated. BAT gene expression from female (E) and male (F) offspring at 15 weeks of age. mRNA levels of *UCP1, PPARg, ApoE, ApoC1, LDLr, LepR, Lep, F2rl1, Il1rn, Nek2, Cenpe, Cdca8, Ccnd1, Cyp26b1, Spp1, Aspg, Akrc19, Fdps, Cyp1a1, Dhrs9, Acsl6, Npc1l1* and *Abhd1* were assessed using RT‐qPCR. The mRNA levels of the selected genes were normalised to *Pgk1*, *Gapdh, Sdha* and *Ppib* housekeeping gene mRNA levels. Number of female offspring: 6 CFD, 7 EmbHFD, 7 HFD, number of male offspring: 6 CFD, 7 EmbHFD, 7 HFD, number of mothers: 6 CFD, 7 EmbHFD and 7 HFD. CFD data (black circles), EmbHFD data (green squares) and HFD data (red triangles) were analysed by multilevel random effects regression and shown as bar graphs with mean ± SEM with individual data points. ^∗^ *p* < 0.05, ^∗∗^ *p* < 0.01, ^∗∗∗^ *p* < 0.001 and ^∗∗∗∗^ *p* < 0.0001 between diet groups.

Gene expression was analysed in offspring BAT through RNAseq and qPCR. In EmbHFD female offspring, 96 genes were downregulated and 120 upregulated compared to CFD. GO and String identified several altered biological functions or pathways involved in metabolic, signalling, cellular and epigenetic processes listed in Figure 4C. In HFD female offspring, 88 genes were downregulated and 77 were upregulated compared with CFD and a similar broad range of biological processes affected as in EmbHFD (listed in Figure 4C). Comparing EmbHFD to HFD female offspring, 34 genes were downregulated and 51 were upregulated, but no biological processes, molecular function or cellular component could be identified.

In male offspring, 14 genes were downregulated and 6 genes upregulated in EmbHFD versus CFD, 1 downregulated and 3 upregulated in HFD vs CFD, and 11 downregulated and 7 upregulated in HFD vs EmbHFD (Figure 4D). No biological processes, molecular function or cellular component were identified in any comparison.

To confirm and consolidate the RNAseq data, qPCR was performed for a selection of genes related to biological functions indicated by RNAseq including thermogenesis (*UCP1*), leptin pathways (*PPARg, LepR, Lep, Ccnd1*), PI3K (*Ccnd1, Lep, F2rl1*) and MapK (*Lep, F2rl1, Il1rn*) signalling; Ppar pathways and cholesterol metabolism (*PPARg, ApoE, ApoC1, Ldlr, LepR, Lep, UCP1, Fdps, Npc1l1, Cyp1a1, Cyp26b1*), angiogenesis and coagulation (*Lep, F2rl1, LepR*), cell metabolic process (*F2rl1, Il1rn, Cenpe, Cdca8, Nek2*), cell division and mitotic pathways (*Ccnd1, Cenpe, Cdca8, Nek2*), steroid metabolic process (*Spp1, Dhrs9, Cyp1a1, Fdps, Akr1c19*) and lipid metabolic process (*Npc1l1, Cyp1a1, Fdps, Akr1c19, Acsl6*).

EmbHFD and HFD offspring females showed a higher expression of *UCP1* and *ApoE* compared to CFD. *Lep* levels were lower in EmbHFD females compared to those in CFD and HFD, confirming RNAseq data. EmbHFD and HFD females had reduced *F2rl1* and *Cenpe* compared to CFD, confirming RNAseq data. EmbHFD females showed lower *Il1rn* expression than CFD. EmbHFD and HFD females showed higher expression of *Akr1c19*, *Fdps* and *Cyp1a1* compared to CF, confirming RNAseq data. HFD females showed lower levels of *Nek2* and *Cdca8* and higher levels of *Npc1l1* compared to CFD, confirming RNAseq data. EmbHFD females showed increased *Spp1* compared to CFD and HFD, similarly to RNAseq data. *Abhd1* levels were lower in EmbHFD females and higher in HFD females, compared to CFD, confirming RNAseq data (Figure 4E).

Upregulation of *UCP1* was found in HFD offspring males and of *Apoe* in both HFD and EmbHFD males, with downregulation of *Ldlr* in both EmbHFD and HFD males, compared to CFD. EmbHFD male offspring showed a higher expression of *Cyp26b1* compared to CFD (Figure 4F).

These data show regulation of BAT expression of several genes involved in lipid metabolism pathways in both HFD and EmbHFD female offspring, with minimal changes and no specific processes or pathways identified in males.

### 3.5. EmbHFD and HFD Offspring Exhibit Deficits in Motor Coordination and HFD Offspring Show Accumulated Muscle Lipid

Behaviour tests were performed on offspring to test locomotion, motor coordination and muscle strength to supplement the energy metabolism data. The IST did not identify differences between groups in muscle strength at age 6 weeks (Figure 5A–B). In the rotarod test assessing balance and motor coordination, HFD females fell more rapidly at age 7 weeks, and both EmbHFD and HFD females fell more rapidly at age 12 weeks compared with CFD (Figure 5C). EmbHFD and HFD males fell more rapidly at age 12 weeks compared with CFD (Figure 5D). Both HFD and EmbHFD males and females declined in their motor outcomes between age 7 and 12 weeks, unlike CFD, showing a combined effect of diet and age in their responsiveness.

**FIGURE 5:**
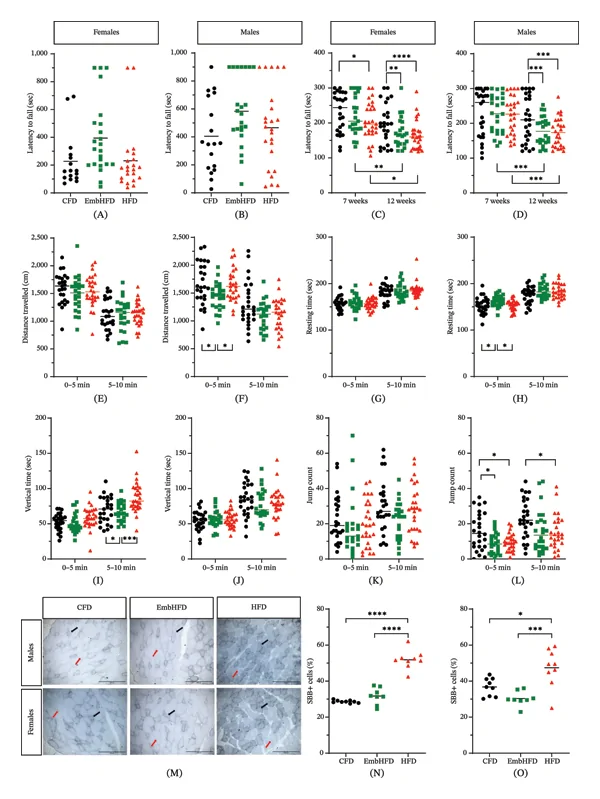
Maternal high‐fat diet affects motor coordination in female and male adult offspring. Inverted screen test latency to fall in the female (A) and male (B) offsprings at 6 weeks old. Number of female offspring: 21 CFD, 23 EmbHFD, 24 HFD, number of male offspring: 20 CFD, 24 EmbHFD, 24 HFD, number of mothers: 6 CFD, 8 EmbHFD and 8 HFD. Rotarod latency to fall for female (C) and male (D) offsprings at 7 and 12 weeks old. Number of female offspring: 26 CFD, 24 EmbHFD, 26 HFD, number of male offspring: 26 CFD, 24 EmbHFD, 26 HFD, number of mothers: 9 CFD, 8 EmbHFD and 9 HFD. A 10‐min open field test of 4‐week‐old offspring was analysed as two periods of 5 min in females (E‐G‐I‐K) and males (F‐H‐J‐L). Distance travelled (E, F), resting time (G, H), vertical time (I, J) and jump counts (K, L) were obtained with the ENV250 software. Number of female offspring: 25 CFD, 23 EmbHFD, 27 HFD, number of male offspring: 26 CFD, 24 EmbHFD, 26 HFD, number of mothers: 9 CFD, 8 EmbHFD and 9 HFD. Representative pictures of Sudan black B–stained sections of mouse offspring gastrocnemius muscle at 15 weeks (M). Cells that showed darker blue are considered as positive (higher lipid content, black arrow) compared to a normal cell (red arrow). Scale bar represents 200 μm. Percentage of Sudan Black B–positive cells (SBB+) in female (N) and male (O) offspring gastrocnemius muscle at 15 weeks. Number of female offspring: 9 CFD, 8 EmbHFD, 9 HFD, number of male offspring: 9 CFD, 8 EmbHFD, 9 HFD, number of mothers: 9 CFD, 8 EmbHFD and 9 HFD. CFD data (black circles), EmbHFD data (green squares) and HFD data (red triangles) were analysed by multilevel random effects regression and shown as dot plots with mean. ^∗^ *p* < 0.05, ^∗∗^ *p* < 0.01, ^∗∗∗^ *p* < 0.001 and ^∗∗∗∗^ *p* < 0.0001 between diet groups or between ages.

In the OFT assessing locomotion at age 4 weeks (Figure 5E–L), EmbHFD male offspring, in either the first (0–5 min) or second (5–10 min) test period, travelled less distance (Figure 5F), rested more (Figure 5H) and jumped less (Figure 5L) than CFD, showing overall lower activity. EmbHFD female offspring spent less time vertically (Figure 5I) and jumped less (Figure 5L). In contrast, the HFD offspring activity was similar to control except for a reduced jump count in males (Figure 5L). EmbHFD and HFD offspring showed no differences in other OFT criteria including entry or time spent in the central area, indicating that anxiety did not contribute to locomotion differences observed (Figure S2). No differences were found when the OFT was performed at age 10 weeks (Figure S2).

Gastrocnemius muscle sections were examined following H&E and Sudan Black B staining for histology and lipid content at age 15 weeks (Figure 5M). No differences in the cross‐sectional area or cell number were evident between diet groups (Figure S3). Sudan Black B revealed more positive cells in HFD, indicating more lipid, but not in EmbHFD female and male sections (Figure 5N–O).

Collectively, EmbHFD and HFD offspring exhibit deficits in motor coordination while EmbHFD offspring also show reduced locomotion and HFD increased muscle lipid accumulation.

## 4. Discussion

This mouse study demonstrates that consumption of maternal HFD, whether restricted to the preimplantation period (EmbHFD) or sustained through gestation and lactation (HFD), induces rapid metabolic changes within the mother that transmit to her developing offspring, causing lifelong physiological, metabolic and behavioural consequences related to health and disease risk. The duration of maternal high fat feeding influences offspring programming, often in a sex‐specific manner, with EmbHFD characterised by high serum leptin (mainly females), elevated SBP, low activity and diminished motor coordination but minimal effects on energy metabolism and muscle lipid composition. While sustained HFD throughout pregnancy and lactation also induced raised serum leptin, high SBP and poor motor coordination in the offspring, this group also experienced excess weight gain during 15 weeks, more severe deficits in RER and EE energy metabolism, reduced relative BAT mass (females) and increased muscle lipid accumulation, yet minimal effects on locomotor activity. What is perhaps most remarkable is that these negative health characteristics across body systems are manifest first in the absence of maternal obesity and second, without the offspring ever eating a HFD themselves.

The above outcomes add to our earlier study using the same mouse model, showing that maternal EmbHFD and HFD caused increasing perturbation in offspring metabolism and brain neural networking and cellular organisation [19]. Collectively, our model therefore substantiates the critical nature of maternal diet during the periconception period as a major pathway influencing offspring development within the context of the DOHaD concept [14–16]. It also demonstrates that maternal preconception healthy lifestyle [36] promoted to avoid the known adverse effects of obesity on offspring [4, 5] may not be sufficient protection unless strict adherence to a healthy diet is maintained. Eating disorders such as binging before or during gestation are well recognised to adversely affect pregnancy outcomes [37]; our mouse data further suggest that transient fatty diet consumption in periconception, when pregnancy would not be confirmed, contributes health risk to the offspring.

The elevated serum leptin levels in offspring exposed to maternal EmbHFD and HFD indicate disruptions in leptin signalling, aligning with previous findings that maternal HFD impairs offspring leptin sensitivity and promotes obesity‐related phenotypes [38]. The lactation period represents a critical window wherein maternal milk, enriched in fat content due to HFD, can amplify metabolic programming by altering lipid transfer to the offspring [39, 40]. Although the observed changes in maternal fatty acid composition were transient, early exposure to altered lipid profiles during key windows may contribute to epigenetic programming and long‐term metabolic effects [41]. Notably, sex‐specific elevations in leptin, particularly in females, may result from oestrogen‐mediated regulation and epigenetic modifications, such as leptin promoter hypomethylation in adipose tissue, which enhances gene transcription [42]. Early leptin surges during preimplantation and lactation periods may impair hypothalamic neurogenesis, disrupting the development of leptin‐responsive neuronal circuits essential for energy balance and glucose homeostasis [43]. This disruption can lead to central leptin resistance, driven by *SOCS3* overexpression and attenuation of the PI3K–Akt signalling pathway, thereby impairing downstream metabolic signalling. Persistent hyperleptinemia in females suggests sex‐dependent epigenetic reprogramming, potentially mediated by differential DNA methylation or histone acetylation in hypothalamic regions, influencing long‐term energy sensing [42].

Increased EE and reduced RER in offspring exposed to maternal HFD suggest a metabolic shift favouring lipid oxidation, primarily mediated through enhanced brown adipose tissue activity. This compensatory thermogenic response, evidenced by increased uncoupling protein 1 (*Ucp1*) gene expression, aligns with findings linking maternal dietary interventions or obesity to offspring thermogenesis [44]. These shifts are sustained into adulthood and are accompanied by BAT‐specific transcriptomic changes indicating both enhanced lipid oxidation and impaired cellular proliferation. Upregulated genes involved in lipid handling and thermogenic function, such as *ApoE* and *Ucp1*, reflect adaptive responses aimed at restoring metabolic homeostasis [45, 46]. However, the concurrent downregulation of genes associated with mitotic regulation and cell cycle progression, such as *Cenpe* and *Cdca8*, implies a reduced regenerative capacity of BAT, potentially limiting long‐term plasticity [47, 48]. Transcriptomic analysis using the same EmbHFD model [19] reported similar systemic outcomes including altered EE and impaired neuromotor performance, further supporting the translational relevance of this developmental model.

While these molecular signatures suggest an initial enhancement in thermogenic activity, the long‐term capacity of BAT to adapt may be constrained by its diminished regenerative potential. These seemingly paradoxical changes suggest that while thermogenic output may initially rise to buffer excess lipid load, impaired BAT turnover may render these tissues less capable of sustained adaptation. Interestingly, male offspring showed minimal transcriptomic alterations despite elevations in *Ucp1* and *ApoE*, highlighting a potential disconnect between transcriptional and functional thermogenesis in males. Female offspring, in contrast, exhibited more pronounced transcriptomic and metabolic changes overall, reinforcing the importance of considering sex as a biological variable in developmental studies [49]. Sex variations may initiate from the preimplantation period with male blastocyst growth, metabolism and gene expression differing from that of females in culture, potentially linked to *X*‐chromosome inactivation delay and differential hormonal sensitivity [50–52]. These early effects may persist into gastrulation and foetal stages [53]. Sex differences may specifically reflect differential sex‐steroid regulation of adipokine and thermogenic pathways: oestrogens can directly enhance leptin expression/secretion, whereas androgens suppress it [54], potentially amplifying programmed hyperleptinemia in females. In parallel, the female‐selective reduction in BAT mass may stem from impaired postnatal BAT expansion/renewal, consistent with our downregulation of mitotic regulators in female BAT and evidence that BAT growth early in life depends on brown adipocyte proliferation [47].

In the current study, offspring of HFD‐fed dams exhibited sustained hyperleptinemia, a phenotype plausibly rooted in epigenetic gene regulation; this is consistent with previous findings observing leptin promoter hypomethylation in adipose tissue, reflecting enhanced leptin gene transcription [55]. Elevated leptin levels are known to activate the sympathetic nervous system, contributing to hypertension and vascular remodelling [55, 56]. Prolonged exposure to elevated leptin levels can disrupt leptin signalling pathways in the brain, leading to central leptin resistance and impaired regulation of thermogenesis, potentially accounting for the unexpected increase in EE despite reduced expression of thermogenic genes in BAT [57].

The motor coordination impairments observed in offspring exposed to an EmbHFD and HFD may indicate systemic effects stemming from altered metabolic and neuroendocrine signalling during development. In addition to its metabolic functions, leptin plays a crucial role in regulating hypothalamus neurogenesis and neuronal migration and maturation during a critical early‐life window [58]. Leptin receptors are also expressed in cerebellar neurons where leptin modulates neuronal signalling relevant to locomotor control [59]. If early leptin signalling is disrupted due to maternal diet, it could hinder the formation of motor circuits [60, 61]. Additionally, abnormal lipid accumulation in the skeletal muscle may negatively affect neuromuscular performance by causing local insulin resistance and mitochondrial stress, as has been documented in both rodent and human studies of lipid overload [62, 63]. Moreover, reduced exploratory behaviour observed in the OFT, particularly among EmbHFD male offsprings, suggests that maternal diet may also influence offspring neurobehavioral development through early‐life stress or altered neurocircuitry. These findings are significant as human epidemiological studies similarly associate maternal obesity and HFDs with an increased risk of metabolic syndrome and neurodevelopmental deficits in their children [7, 64, 65]. The perinatal period represents a critical intervention window during which targeted maternal dietary modification could improve offspring health outcomes and reduce the intergenerational transmission of metabolic disease [5].

This study highlights the impact of a maternal EmbHFD and HFD on the metabolism of offspring, although it has certain limitations. We did not assess the influence of the maternal microbiome, the diet’s effects post‐weaning or long‐term motor coordination outcomes. Moreover, we are aware that the results observed may be specific to the murine strain and diet used. Future research should integrate long‐term monitoring to gain better insights into the timing and reversibility of programming effects caused by these diet treatments. In conclusion, exposure to a high‐fat maternal diet modifies the metabolic physiology of offspring through hormonal and tissue‐specific changes that persist into adulthood. These results reinforce the core principles of the DOHaD concept and emphasise the substantial impact of maternal diet on the long‐term health of the offspring. Our findings highlight the critical need for early preventive strategies aimed at improving maternal nutrition to alleviate the intergenerational transmission of metabolic disease.

## Author Contributions

Conceptualisation: Tom P. Fleming, Judith J. Eckert, Neil R. Smyth and Sandrine Willaime‐Morawek; formal analysis: Irene Peral‐Sanchez and Eda Sezer; investigation: Irene Peral‐Sanchez, Eda Sezer and Rachel Morris; resources: Patrick C. McHugh, Jeanette L. Norman and Jon Ward; writing–original draft: Diego A. Ojeda, Tom P. Fleming and Sandrine Willaime‐Morawek; writing–review and editing; Irene Peral‐Sanchez, Eda Sezer, Diego A. Ojeda, Philip C. Calder, Patrick C. McHugh, Rachel Morris, Jeanette L. Norman, Jon Ward, Tom P. Fleming, Judith J. Eckert, Neil R. Smyth and Sandrine Willaime‐Morawek; visualisation: Irene Peral‐Sanchez, Eda Sezer and Sandrine Willaime‐Morawek; funding acquisition: Eda Sezer, Diego A. Ojeda, Tom P. Fleming, Judith J. Eckert, Neil R. Smyth and Sandrine Willaime‐Morawek.

## Funding

This project was funded by the EU’s HORIZON2020 Marie‐Curie Skłodowska Curie Grant (DohART‐NET Periconceptional Programming of Health Training Network, under grant agreement 812660), the Turkish Ministry of National Education‐1414 YLSY PhD Bursary Programme, the Faculty of Medicine, University of Southampton, the Biotechnology and Biological Sciences Research Council (BBSRC) (BB/F007450/1) and the Colciencias Doctoral Scholarship Colombia (Convocatoria 646‐19 Doctorados en el Exterior).

## Conflicts of Interest

The authors declare no conflicts of interest.

## Supporting Information

Additional supporting information can be found online in the Supporting Information section.

## Supporting information


**Supporting Information 1** **Supporting File 1:** Maternal high‐fat diet does not affect maternal serum fatty acid concentrations permanently nor offspring serum fatty acid concentration. Fatty acid content of lipid from TAG, CE, NEFA, PC serum of mothers at G3.5 and L21, from TAG, CE, NEFA, PC serum of male and female offspring at 15 weeks. Significance with *p* < 0.05 shaded in blue (lower than CFD) or red (higher than CFD), *n* = 5 per group.

**Supporting Information 2** **Supporting Figure 2:** Maternal high‐fat diet does not affect motor coordination at 10 weeks nor anxiety at 4 and 10 weeks. A 10‐min open field test of 4‐week‐old and 10‐week‐old offspring was analysed as two periods of 5 min in females and males. Vertical counts, ambulatory count, average velocity, jump time, distance travelled, resting time, vertical time, jump counts, entry times to central area, time spent in central area and time spent in residual area were obtained with the ENV250 software.

**Supporting Information 3** **Supporting Figure 3:** Maternal high‐fat diet does not affect muscle morphology or cell number in offspring. Representative haematoxylin and eosin muscle sections of mouse offspring GA muscle in the different diet groups. Cross‐sectional area (CSA) in male and female offspring at 15 weeks of age. Cell numbers per section in male and female offspring at 15 weeks of age.

## Data Availability

The data that support the findings of this study are openly available in the Pure University of Southampton at https://pure.soton.ac.uk/, reference numbers https://doi.org/10.5258/SOTON/D2904 and https://doi.org/10.5258/SOTON/D3117.

## References

[1] Singh B. K. , Mishra S. R. , and Khatri R. B. , Trends and Determinants of Clustering for Non-Communicable Disease Risk Factors in Women of Reproductive Age in Nepal, PLoS One. (2024) 19, no. 10, 10.1371/journal.pone.0309322. PMC11446422 39356704

[2] Yadav I. , Jyoti S. , Bahik C. , Acharya J. , Bohaju A. , and Yadav S. K. , Non-Communicable Diseases Among Women of Reproductive Age Visiting the Department of Obstetrics and Gynecology of a Tertiary Care Hospital, JNMA J Nepal Med Assoc. (2024) 62, no. 270, 95–98, 10.31729/jnma.8471. PMC10924513 38409969

[3] Firoz T. , Pineles B. , Navrange N. , Grimshaw A. , Oladapo O. , and Chou D. , Non-Communicable Diseases and Maternal Health: A Scoping Review, BMC Pregnancy and Childbirth. (2022) 22, no. 1, 10.1186/s12884-022-05047-6. PMC9587654 36273124

[4] Denizli M. , Capitano M. L. , and Kua K. L. , Maternal Obesity and the Impact of Associated Early-Life Inflammation on Long-Term Health of Offspring, Frontiers in Cellular and Infection Microbiology. (2022) 12, 10.3389/fcimb.2022.940937. PMC9523142 36189369

[5] Godfrey K. M. , Reynolds R. M. , Prescott S. L. et al., Influence of Maternal Obesity on the Long-Term Health of Offspring, Lancet Diabetes and Endocrinology. (2017) 5, no. 1, 53–64, 10.1016/S2213-8587(16)30107-3. PMC5245733 27743978

[6] Urbonaite G. , Knyzeliene A. , Bunn F. S. , Smalskys A. , and Neniskyte U. , The Impact of Maternal High-Fat Diet on Offspring Neurodevelopment, Frontiers in Neuroscience. (2022) 16, 10.3389/fnins.2022.909762. PMC9354026 35937892

[7] Yang Z. , Feng G. , Gao X. et al., Maternal Adiposity and Perinatal and Offspring Outcomes: An Umbrella Review, Nature Human Behaviour. (2024) 8, no. 12, 2406–2422, 10.1038/s41562-024-01994-6. 39394444

[8] Menting M. D. , Mintjens S. , van de Beek C. et al., Maternal Obesity in Pregnancy Impacts Offspring Cardiometabolic Health: Systematic Review and Meta-Analysis of Animal Studies, Obesity Reviews. (2019) 20, no. 5, 675–685, 10.1111/obr.12817. PMC6849816 30633422

[9] Samuelsson A. M. , Matthews P. A. , Argenton M. et al., Diet-Induced Obesity in Female Mice Leads to Offspring Hyperphagia, Adiposity, Hypertension, and Insulin Resistance: a Novel Murine Model of Developmental Programming, Hypertension. (2008) 51, no. 2, 383–392, 10.1161/HYPERTENSIONAHA.107.101477. 18086952

[10] Saullo C. M. , Sinzato Y. K. , Paula V. G. et al., Exposure to Maternal Hyperglycemia and High-Fat Diet Consumption After Weaning in Rats: Repercussions on Periovarian Adipose Tissue, J Dev Orig Health Dis. (2022) 13, no. 5, 634–641, 10.1017/S2040174421000672. 34859760

[11] Yang Z. , Jiang J. , Chen M. et al., Sex-Specific Effects of Maternal and Post-Weaning High-Fat Diet on Adipose Tissue Remodeling and Asprosin Expression in Mice Offspring, Molecular Nutrition & Food Research. (2022) 66, no. 4, 10.1002/mnfr.202100470. 34933410

[12] Barker D. J. , The Origins of the Developmental Origins Theory, Journal of Internal Medicine. (2007) 261, no. 5, 412–417, (in eng) 10.1111/j.1365-2796.2007.01809.x. 17444880

[13] Hanson M. A. and Gluckman P. D. , Early Developmental Conditioning of Later Health and Disease: Physiology or Pathophysiology?, Physiological Reviews. (2014) 94, no. 4, 1027–1076, 10.1152/physrev.00029.2013. PMC4187033 25287859

[14] Fleming T. P. , Watkins A. J. , Velazquez M. A. et al., Origins of Lifetime Health Around the Time of Conception: Causes and Consequences, Lancet. (2018) 391, no. 10132, 1842–1852, 10.1016/S0140-6736(18)30312-X. PMC5975952 29673874

[15] Velazquez M. A. , Idriss A. , Chavatte-Palmer P. , and Fleming T. P. , The Mammalian Preimplantation Embryo: Its Role in the Environmental Programming of Postnatal Health and Performance, Animal Reproduction Science. (2023) 256, 10.1016/j.anireprosci.2023.107321. 37647800

[16] Peral-Sanchez I. , Hojeij B. , Ojeda D. A. , Steegers-Theunissen R. P. M. , and Willaime-Morawek S. , Epigenetics in the Uterine Environment: How Maternal Diet and ART May Influence the Epigenome in the Offspring With Long-Term Health Consequences, Genes. (2021) 13, no. 1, (in eng) 10.3390/genes13010031. PMC8774448 35052371

[17] Canovas S. and Ross P. J. , Epigenetics in Preimplantation Mammalian Development, Theriogenology. (2016) 86, no. 1, 69–79, 10.1016/j.theriogenology.2016.04.020. PMC4885810 27165992

[18] Gretchev A. , Temples H. S. , England-Mason G. et al., Unpacking Developmental Programming: A Conceptual and Historical Analysis in the Context of Dohad, Journal of Developmental Origins of Health and Disease. (2026) 17, 10.1017/S2040174426100518. 41919437

[19] Ojeda D. A. , Hutton O. , Hopkins R. et al., Preimplantation or Gestation/Lactation High-Fat Diet Alters Adult Offspring Metabolism and Neurogenesis, Brain Commun. (2023) 5, no. 2, 10.1093/braincomms/fcad093. PMC10077335 37033334

[20] Chen L. W. , Loy S. L. , Tint M. T. et al., Maternal Pregnancy Diet Quality, Night Eating, and Offspring Metabolic Health: the GUSTO Study, Pediatric Research. (2025) 97, no. 5, 1528–1536, 10.1038/s41390-024-03574-w. 39300274

[21] Deacon R. M. , Measuring the Strength of Mice, Journal of Visualized Experiments: JoVE. (2013) 76, 10.3791/2610. PMC3725666 23770643

[22] Tam C. S. , Power J. E. , Markovic T. P. et al., The Effects of High-Fat Feeding on Physical Function and Skeletal Muscle Extracellular Matrix, Nutrition & Diabetes. (2015) 5, no. 12, 10.1038/nutd.2015.39. PMC4735053 26657013

[23] Deacon R. M. , Measuring Motor Coordination in Mice, Journal of Visualized Experiments: JoVE. (2013) 75, 10.3791/2609. PMC3724562 23748408

[24] Zhang Y. , Fischer K. E. , Soto V. et al., Obesity-Induced Oxidative Stress, Accelerated Functional Decline with Age and Increased Mortality in Mice, Archives of Biochemistry and Biophysics. (2015) 576, 39–48, 10.1016/j.abb.2014.12.018. PMC4456198 25558793

[25] Fisk H. L. , West A. L. , Childs C. E. , Burdge G. C. , and Calder P. C. , The Use of Gas Chromatography to Analyze Compositional Changes of Fatty Acids in Rat Liver Tissue During Pregnancy, Journal of Visualized Experiments: JoVE. (2014) 85, 10.3791/51445. PMC4151317 24686562

[26] Kumar A. , Accorsi A. , Rhee Y. , and Girgenrath M. , Do’s and Dont’s in the Preparation of Muscle Cryosections for Histological Analysis, Journal of Visualized Experiments: JoVE. (2015) no. 99, 10.3791/52793. PMC4542757 26066009

[27] Yang Y. , Li P. , Lin Y. et al., Gene Expression Profiling of the Liver and Lung in Mice After Exposure to Zno Quantum Dots, International Journal of Nanomedicine. (2020) 15, 2947–2955, 10.2147/IJN.S246754. PMC7196207 32425526

[28] Livak K. J. and Schmittgen T. D. , Analysis of Relative Gene Expression Data Using Real-Time Quantitative PCR and the 2(-Delta Delta C(T)) Method, Methods. (2001) 25, no. 4, 402–408, 10.1006/meth.2001.1262. 11846609

[29] Hellemans J. , Mortier G. , De Paepe A. , Speleman F. , and Vandesompele J. , Qbase Relative Quantification Framework and Software for Management and Automated Analysis of Real-Time Quantitative PCR Data, Genome Biology. (2007) 8, no. 2, 10.1186/gb-2007-8-2-r19. PMC1852402 17291332

[30] Xie Q. , Gu X. , Chen J. et al., Soyasaponins Reduce Inflammation and Improve Serum Lipid Profiles and Glucose Homeostasis in High Fat Diet-Induced Obese Mice, Molecular Nutrition & Food Research. (2018) 62, no. 19, 10.1002/mnfr.201800205. 30070430

[31] Bernardis L. L. and Patterson B. D. , Correlation Between ‘Lee Index’ and Carcass Fat Content in Weanling and Adult Female Rats With Hypothalamic Lesions, Journal of Endocrinology. (1968) 40, no. 4, 527–528, 10.1677/joe.0.0400527. 4868415

[32] Song Y. P. , Chen Y. H. , Gao L. et al., Differential Effects of High-Fat Diets Before Pregnancy and/or During Pregnancy on Fetal Growth Development, Life Sciences. (2018) 212, 241–250, 10.1016/j.lfs.2018.10.008. 30300654

[33] Peral Sanchez I. , The Effect of High-Fat Diet During Mouse Preimplantation and Pregnancy-Lactation on Uterine Fluid Protein Composition, Maternal Metabolism and Offspring Health, 2023, University of Southampton, https://eprints.soton.ac.uk/485790/, [Online]. Available.

[34] Sezer E. , The Impact of Maternal High Fat Diet on Offspring Brain, Behaviour and Cognitive Functions, 2024, University of Southampton, Southampton, Doctoral Thesis https://eprints.soton.ac.uk/id/eprint/491140/.

[35] Kulterer O. C. , Niederstaetter L. , Herz C. T. et al., The Presence of Active Brown Adipose Tissue Determines Cold-Induced Energy Expenditure and Oxylipin Profiles in Humans, Journal of Clinical Endocrinology and Metabolism. (2020) 105, no. 7, 2203–2216, 10.1210/clinem/dgaa183. 32343312

[36] Stephenson J. , Heslehurst N. , Hall J. et al., Before the Beginning: Nutrition and Lifestyle in the Preconception Period and Its Importance for Future Health, Lancet. (2018) 391, no. 10132, 1830–1841, 10.1016/S0140-6736(18)30311-8. PMC6075697 29673873

[37] Milembamane M. M. , Moussa N. M. M. , Twynstra J. P. , and Seabrook J. A. P. , Maternal Eating Disorders and Adverse Birth Outcomes: A Systematic Review and Meta-Analysis, Canadian Journal of Dietetic Practice and Research. (2024) 85, no. 1, 45–53, 10.3148/cjdpr-2023-019. 38032141

[38] Sun B. , Purcell R. H. , Terrillion C. E. , Yan J. , Moran T. H. , and Tamashiro K. L. , Maternal High-Fat Diet During Gestation or Suckling Differentially Affects Offspring Leptin Sensitivity and Obesity, Diabetes. (2012) 61, no. 11, 2833–2841, 10.2337/db11-0957. PMC3478561 22751689

[39] Carlson Z. , Hafner H. , El Habbal N. et al., Short Term Changes in Dietary Fat Content and Metformin Treatment During Lactation Impact Milk Composition and Mammary Gland Morphology, Journal of Mammary Gland Biology and Neoplasia. (2022) 27, no. 1, 1–18, 10.1007/s10911-022-09512-y. PMC12762465 35137304

[40] Kim K. , Varghese M. , Sun H. et al., The Influence of Maternal High Fat Diet During Lactation on Offspring Hematopoietic Priming, Endocrinology. (2023) 165, no. 1, 10.1210/endocr/bqad182. PMC11032250 38048597

[41] Tang S. , Wu H. , Chen Q. et al., Maternal Obesity Induces the Meiotic Defects and Epigenetic Alterations During Fetal Oocyte Development, Advanced Science (Weinheim). (2024) 11, no. 30, 10.1002/advs.202309184. PMC11321662 38868907

[42] Muller L. , Oelkrug R. , Mittag J. et al., Sex-Specific Role of Epigenetic Modification of a Leptin Upstream Enhancer in Adipose Tissue, Clinical Epigenetics. (2025) 17, no. 1, 10.1186/s13148-025-01830-2. PMC11816557 39934931

[43] Xu Y. , Yang D. , Wang L. et al., Maternal High Fat Diet in Lactation Impacts Hypothalamic Neurogenesis and Neurotrophic Development, Leading to Later Life Susceptibility to Obesity in Male but Not Female Mice, Advanced Science (Weinheim). (2023) 10, no. 35, 10.1002/advs.202305472. PMC10724448 37867217

[44] Hua L. , Shen W. , Gao D. et al., Maternal Obesity Impairs Fetal Brown Adipogenesis by Attenuating Fetal FGF21 Signaling, Cell Reports. (2025) 44, no. 7, 10.1016/j.celrep.2025.115935. 40591460

[45] Savva C. , Helguero L. A. , González-Granillo M. et al., Maternal High-Fat Diet Programs White and Brown Adipose Tissue Lipidome and Transcriptome in Offspring in a Sex- and Tissue-Dependent Manner in Mice, International Journal of Obesity. (2022) 46, no. 4, 831–842, 10.1038/s41366-021-01060-5. PMC8960419 34997206

[46] Liang X. , Yang Q. , Zhang L. et al., Maternal High-Fat Diet During Lactation Impairs Thermogenic Function of Brown Adipose Tissue in Offspring Mice, Scientific Reports. (2016) 6, no. 1, 10.1038/srep34345. PMC5043374 27686741

[47] Negron S. G. , Ercan-Sencicek A. G. , Freed J. , Walters M. , and Lin Z. , Both Proliferation and Lipogenesis of Brown Adipocytes Contribute to Postnatal Brown Adipose Tissue Growth in Mice, Scientific Reports. (2020) 10, no. 1, 10.1038/s41598-020-77362-x. PMC7683731 33230135

[48] Lopez-Mejia I. C. , Castillo-Armengol J. , Lagarrigue S. , and Fajas L. , Role of Cell Cycle Regulators in Adipose Tissue and Whole Body Energy Homeostasis, Cellular and Molecular Life Sciences. (2018) 75, no. 6, 975–987, 10.1007/s00018-017-2668-9. PMC11105252 28988292

[49] Alsiraj Y. , Huang H. , Shoemaker R. et al., Maternal Nutritional Programming: Sex-Specific Cardiovascular and Immune Outcomes Following Perinatal High-Fat Diet Exposure, Nutrients. (2025) 17, no. 9, 10.3390/nu17091464. PMC12073119 40362773

[50] Valdivia R. P. , Kunieda T. , Azuma S. , and Toyoda Y. , PCR Sexing and Developmental Rate Differences in Preimplantation Mouse Embryos Fertilized and Cultured in Vitro, Molecular Reproduction and Development. (1993) 35, no. 2, 121–126, 10.1002/mrd.1080350204. 8318217

[51] Gardner D. K. , Larman M. G. , and Thouas G. A. , Sex-Related Physiology of the Preimplantation Embryo, Molecular Human Reproduction. (2010) 16, no. 8, 539–547, 10.1093/molehr/gaq042. 20501630

[52] Shi M. , Li G. , Araujo H. M. et al., Sex-Biased Transcriptome in in Vitro Produced Bovine Early Embryos, Cell & Bioscience. (2025) 15, no. 1, 10.1186/s13578-025-01459-x. PMC12392601 40866999

[53] Lu Y. , Qin M. , Qi X. et al., Sex Differences in Human Pre-Gastrulation Embryos, Science China Life Sciences. (2025) 68, no. 2, 397–415, 10.1007/s11427-024-2721-y. 39327393

[54] Jenks M. Z. , Fairfield H. E. , Johnson E. C. , Morrison R. F. , and Muday G. K. , Sex Steroid Hormones Regulate Leptin Transcript Accumulation and Protein Secretion in 3T3-L1 Cells, Scientific Reports. (2017) 7, no. 1, 10.1038/s41598-017-07473-5. PMC5558017 28811502

[55] Lin X.-H. , Gao L. , Tian S. et al., Maternal High-Fat-Diet Exposure is Associated With Elevated Blood Pressure and Sustained Increased Leptin Levels Through Epigenetic Memory in Offspring, Scientific Reports. (2021) 11, no. 1, 10.1038/s41598-020-79604-4. PMC7801715 33431976

[56] Tain Y.-L. and Hsu C.-N. , Maternal High-Fat Diet and Offspring Hypertension, International Journal of Molecular Sciences. (2022) 23, no. 15, https://www.mdpi.com/1422-0067/23/15/8179, [Online]. Available 10.3390/ijms23158179. PMC9332200 35897755

[57] Knight Z. A. , Hannan K. S. , Greenberg M. L. , and Friedman J. M. , Hyperleptinemia Is Required for the Development of Leptin Resistance, PLoS One. (2010) 5, no. 6, (in eng) 10.1371/journal.pone.0011376. PMC2894068 20613882

[58] Bouret S. G. , Draper S. J. , and Simerly R. B. , Trophic Action of Leptin on Hypothalamic Neurons that Regulate Feeding, Science. (2004) 304, no. 5667, 108–110, 10.1126/science.1095004. 15064420

[59] Irving A. J. , Wallace L. , Durakoglugil D. , and Harvey J. , Leptin Enhances NR2B-mediated N-Methyl-D-Aspartate Responses via a Mitogen-Activated Protein Kinase-Dependent Process in Cerebellar Granule Cells, Neuroscience. (2006) 138, no. 4, 1137–1148, 10.1016/j.neuroscience.2005.11.042. PMC1613257 16413128

[60] Ahima R. S. , Bjorbaek C. , Osei S. , and Flier J. S. , Regulation of Neuronal and Glial Proteins by Leptin: Implications for Brain Development, Endocrinology. (1999) 140, no. 6, 2755–2762, 10.1210/endo.140.6.6774. 10342866

[61] Bouret S. G. , Neurodevelopmental Actions of Leptin, Brain Research. (2010) 1350, 2–9, 10.1016/j.brainres.2010.04.011. PMC3654158 20399755

[62] Corcoran M. P. , Lamon-Fava S. , and Fielding R. A. , Skeletal Muscle Lipid Deposition and Insulin Resistance: Effect of Dietary Fatty Acids and Exercise, American Journal of Clinical Nutrition. (2007) 85, no. 3, 662–677, (in eng) 10.1093/ajcn/85.3.662. 17344486

[63] Anderson E. J. , Lustig M. E. , Boyle K. E. et al., Mitochondrial H_2_O_2_ Emission and Cellular Redox State Link Excess Fat Intake to Insulin Resistance in Both Rodents and Humans, Journal of Clinical Investigation. (2009) 119, no. 3, 573–581, (in eng) 10.1172/jci37048. PMC2648700 19188683

[64] Krakowiak P. , Walker C. K. , Bremer A. A. et al., Maternal Metabolic Conditions and Risk for Autism and Other Neurodevelopmental Disorders, Pediatrics. (2012) 129, no. 5, e1121–e1128, 10.1542/peds.2011-2583. PMC3340592 22492772

[65] Boney C. M. , Verma A. , Tucker R. , and Vohr B. R. , Metabolic Syndrome in Childhood: Association With Birth Weight, Maternal Obesity, and Gestational Diabetes Mellitus, Pediatrics. (2005) 115, no. 3, e290–e296, 10.1542/peds.2004-1808. 15741354

