# Supplementary material for: Maternal High‐Fat Diet, During Preimplantation or Gestation/Lactation, Alters Mouse Offspring Metabolic, Molecular and Locomotor Phenotypes
Source: J Nutr Metab. 2026 Sep 30;2026:5286440. doi: 10.1155/jnme/5286440 (PMC13625825; doi:10.1155/jnme/5286440)

Supplementary S2

Open Field Test 1 at age 4 weeks

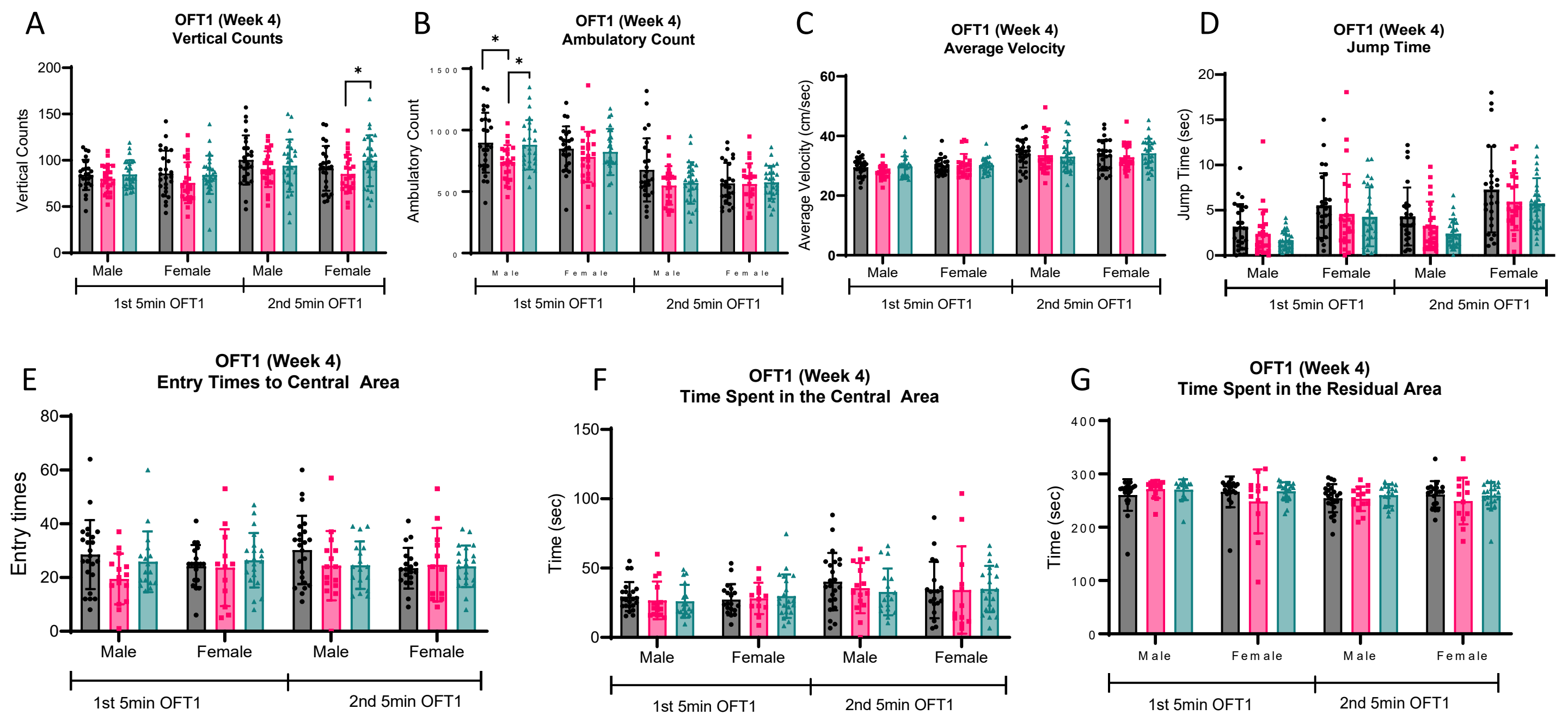

Open Field Test 2 at age 10 weeks

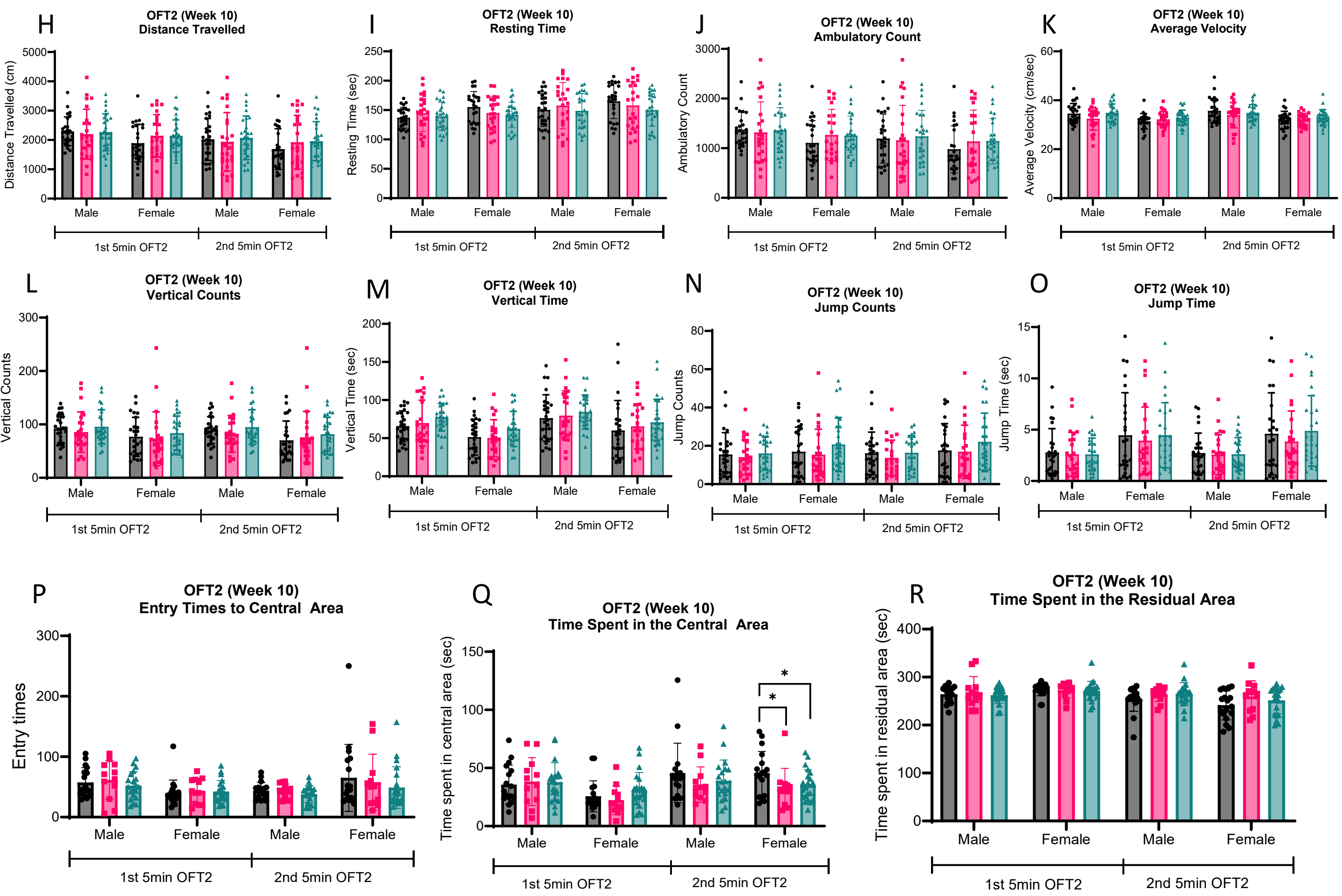

Supplement: Supplementary file 1 [file JNME-2026-5286440-s001.pdf]
