# Supplementary material for: Maternal High‐Fat Diet, During Preimplantation or Gestation/Lactation, Alters Mouse Offspring Metabolic, Molecular and Locomotor Phenotypes
Source: J Nutr Metab. 2026 Sep 30;2026:5286440. doi: 10.1155/jnme/5286440 (PMC13625825; doi:10.1155/jnme/5286440)
Supplement: Supplementary file 2 [file JNME-2026-5286440-s003.pdf]

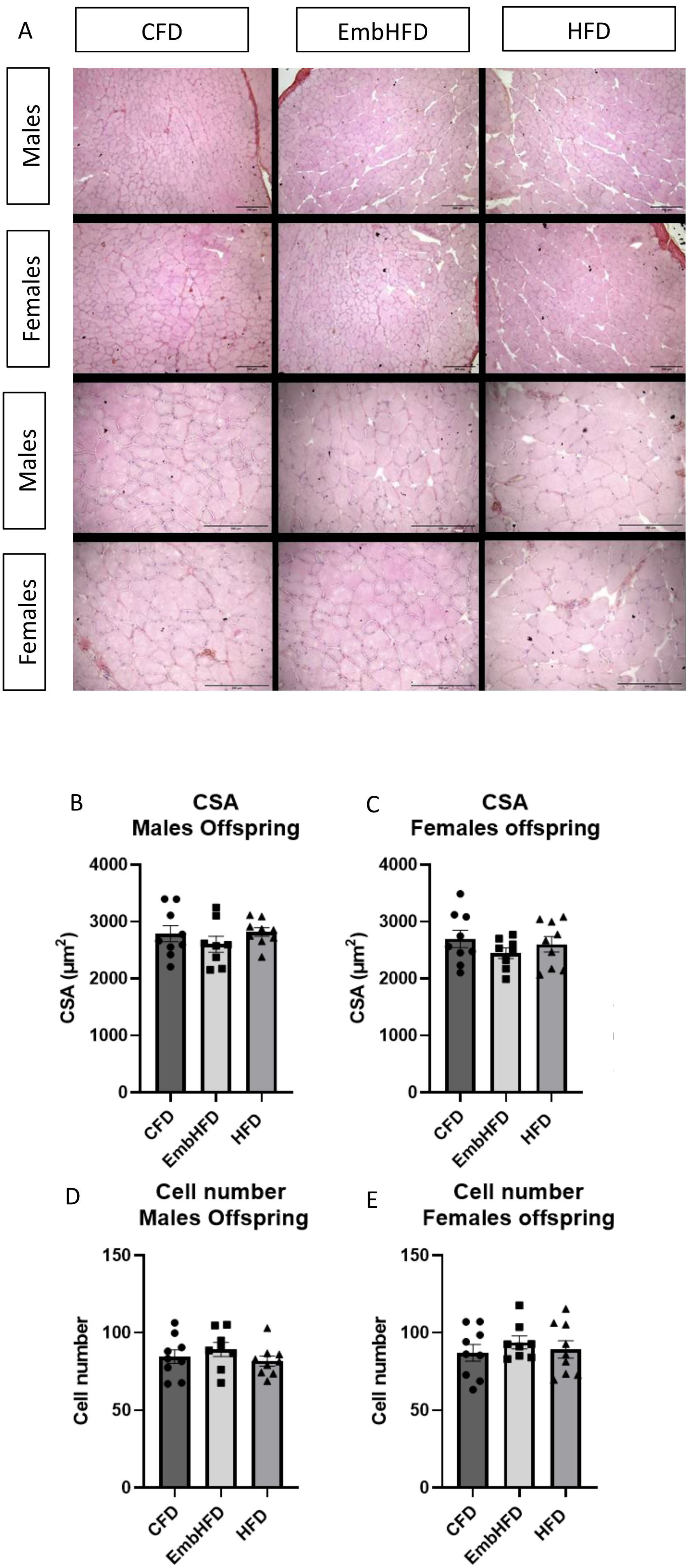
A
CFD
EmbHFD
HFD
Males
Females
Males
Females
B
CSA
Males Offspring
CSA (μm²)
4000
3000
2000
1000
0
CFD
EmbHFD
HFD
C
CSA
Females offspring
CSA (μm²)
4000
3000
2000
1000
0
CFD
EmbHFD
HFD
D
Cell number
Males Offspring
Cell number
150
100
50
0
CFD
EmbHFD
HFD
E
Cell number
Females offspring
Cell number
150
100
50
0
CFD
EmbHFD
HFD
